# Depression and anxiety mediate the relationship between COVID-19 stay-at-home orders and tobacco and marijuana use

**DOI:** 10.1371/journal.pone.0337996

**Published:** 2025-12-05

**Authors:** Geoffrey Carney-Knisely, Jolynn Pek, Amy K. Ferketich, Tasleem J. Padamsee, Tamar Gur, Parvati Singh

**Affiliations:** 1 Division of Epidemiology, The Ohio State University College of Public Health, Columbus, Ohio, United States of America; 2 Department of Psychology, The Ohio State University College of Arts and Sciences, Columbus, Ohio, United States of America; 3 Division of Health Services Management and Policy, The Ohio State University College of Public Health, Columbus, Ohio, United States of America; 4 Department of Psychiatry and Behavioral Health, The Ohio State University College of Medicine, Columbus, Ohio, United States of America; Donald and Barbara Zucker School of Medicine at Hofstra/Northwell, UNITED STATES OF AMERICA

## Abstract

**Background:**

The COVID-19 pandemic had notable impacts on the mental health of the U.S. population. There were concerns about how the pandemic affected substance use in the population. The overall objective of this study was to assess whether COVID-19 Stay-At-Home (SAH) orders, an ambient ecological stressor, as well as the severity of depressive and anxious symptoms, can explain tobacco and marijuana use.

**Methods:**

Data come from the first seven waves of the Understanding America Study, a nationally representative longitudinal web-based panel study. A total of 7,554 persons participated in the first seven waves, resulting in 43,582 observations. Cigarette use as a measure was not included until wave four of the study; 7,034 persons participated in waves four through seven, resulting in 24,893 observations. The primary outcomes were self-reported past seven-day use of cigarette products and past seven-day use of marijuana products. Self-reported depressive and anxious symptom severity, the proposed mediator, was measured using the Patient Health Questionnaire-4 (PHQ-4). The primary exposure was a binary indicator for the presence of an SAH order. All variables were measured biweekly. Generalized Estimating Equations (GEE) were used to assess single-mediator models.

**Results:**

Persons under SAH orders had 2.18 (95% CI: 1.27, 3.73) times the odds of moderate-to-severe depression across the first seven waves, relative to those living in states without SAH orders. Those with moderate-to-severe depression and anxiety had lower odds of both marijuana (OR = 0.37, 95% CI: 0.17, 0.84) and cigarette use (OR = 0.29, 95% CI: 0.13, 0.65) compared to those with normal-mild PHQ-4 scores. Worsened mental health within a person resulted in 0.22 (95% CI: 0.12, 0.40) times the odds of marijuana use and 0.26 (95% CI: 0.15, 0.47) times the odds of cigarette use. Tests of the joint effects suggest evidence of multiple mediated pathways.

## Introduction

In March 2020, after the United States government declared a national emergency due to the COVID-19 pandemic, state governments implemented various “lockdown” measures to mitigate infection from the virus. These policies included Stay-At-Home (SAH) orders, which encompassed shelter-in-place orders, gathering restrictions, and business and school closures [1,2]. Seven states did not implement formal SAH orders during the public health emergency [2].

Early in the pandemic, experts raised concerns about the effect of SAH policies on the mental health and substance use of affected populations [3–5]. Between April and June 2020, nearly 41% of U.S. adults reported a negative mental or behavioral health condition [6]. Further, there was an increase in the prevalence of depression and anxiety during those months compared to the same period in 2019 [6]. Research on consumer behavior suggested that sales of alcohol, tobacco, and marijuana increased during the pandemic, particularly among young adults and higher-income households [7,8].

The core hypothesis of this study is that COVID-19 restrictions, particularly SAH orders, act as a significant social stressor, leading to psychological distress. This distress is hypothesized to mediate changes in substance use, as individuals may turn to substances such as tobacco and marijuana to cope. Previous ecological stressors, including pandemics, natural disasters, and terrorist attacks, suggest the potential for long-term effects on mental health and substance use [9–12]. Under the self-medication hypothesis, persons may use substances to ameliorate distressing psychological symptoms, not specific to psychiatric conditions [13–16]. Persons choose substances based on how they perceive the substance will affect them, not its physiologic effect. Perceptions of stress relief are well-described motivators for smoking tobacco [17,18]. People in the U.S. may use marijuana to relieve depressive or anxious symptoms [19–21]. Conversely, it is plausible that macrosocial stressors induce population-level “inhibition effects” wherein healthful behaviors may increase, and consumption of harmful substances may decline as a risk-averse response to economic uncertainty [22–24].

While previous work identified cross-sectional associations between COVID-19 policies, mental health, and substance use, limited research has focused on longitudinal changes during the initial period of the pandemic. A limitation of cross-sectional studies is a lack of temporal precedence. We hypothesize that the impact of SAH orders on mental health is temporally prior to and linked with changes in substance use. Current longitudinal literature focuses on changes in mental health or substance use in isolation [25–29]. We address these limitations by estimating temporal relationships between COVID-19 SAH orders and both mental health and substance-use outcomes. Further, we describe how changes within a person’s mental health relate to tobacco use and marijuana use outcomes within a single system of simultaneous equations. Thus, the goal of this research is to describe and understand a mediation mechanism by which exposure to SAH orders results in changes in mental health, which in turn influence tobacco and marijuana use.

## Methods

### Settings and participants

Details about the survey used in this study can be found elsewhere [30]. Briefly, the Understanding America Study (UAS) is a nationally representative longitudinal web-based panel study of persons aged 18 and older. Participants were considered eligible if the respondent in a contacted household was at least 18 years old [31]. Participants were recruited using Address Based Sampling from a random national sample. The UAS COVID-19 tracking survey, launched on March 10, 2020, focused on attitudes and behaviors concerning the coronavirus pandemic. Using the complete UAS sample as a base, the tracking survey has completion rates between 67.1% and 80.4% [32]. The first seven waves of the COVID-19 tracking survey were used for this analysis for a total of 43,582 observations from 7,554 persons. Cigarette use was not assessed in the first three waves. For analyses including cigarette use, only waves four through seven were used resulting in 24,893 observations from 7,034 persons.

### Procedures

The UAS COVID-19 surveys were developed internally by the University of Southern California [30]. Panel members were randomized to respond on a pre-determined day of the week such that all panel members were invited to complete the tracking survey within a 14-day period; they received an incentive only for completing the survey on their assigned day. All surveys were conducted online.

### Measures

#### Dependent variables.

Two outcome measures were assessed as dependent variables of interest. First, An indicator for current smoking status. This variable was determined using the item “*the number of days in the past week smoked all or part of a cigarette.”* Second, an indicator of current marijuana use was determined using the item “*the number of days in the past week used cannabis products.*” Any non-zero response indicated current use for both measures.

The proposed mediator is the severity of mental health symptoms, measured using the Patient Health Questionnaire-4 (PHQ-4). The PHQ-4 is a four-item metric consisting of two two-item ultra-brief screening tools for depression and anxiety. Scores range from zero to 12. In U.S. populations, the scale has demonstrated strong reliability and validity in measuring depression and anxiety [33–35]. Using scoring criteria, PHQ-4 score was dichotomized into categories of normal-to-mild and moderate-to-severe [33].

#### Independent variables.

The primary exposure was the presence of COVID-19 SAH orders. This was measured at the state-level using policy information from the University of Washington COVID-19 Policy Center [36]. The SAH variable was a dichotomous indicator based on whether a respondent lived in a state that implemented a SAH order and was under a SAH order at the time of survey completion. Respondents were only considered exposed if the survey was completed between the start and end dates of a SAH order within a specific state. Repeated measures were nested within respondents, and respondents were nested within states. There are, however, two sources of spatial-temporal variation within this indicator: 1) within a state as previously unexposed respondents became exposed to a SAH order, and 2) between states, as states enacted different types of SAH orders for different lengths. Seven states never implemented SAH orders: Arkansas, Iowa, Nebraska, North Dakota, South Dakota, Utah, and Wyoming.

#### Covariates.

Covariates included age (years), gender (dichotomous male/female), race (White Non-Hispanic, Black Non-Hispanic, Asian, and other), ethnicity (dichotomous indicator of Hispanic/Latino), educational attainment (less than a high-school education, high school degree or equivalent, college graduate or higher), and a binary indicator of a state’s marijuana legalization status to account for increased availability of marijuana in states with legalization.

### Statistical analysis

**Fig 1:**
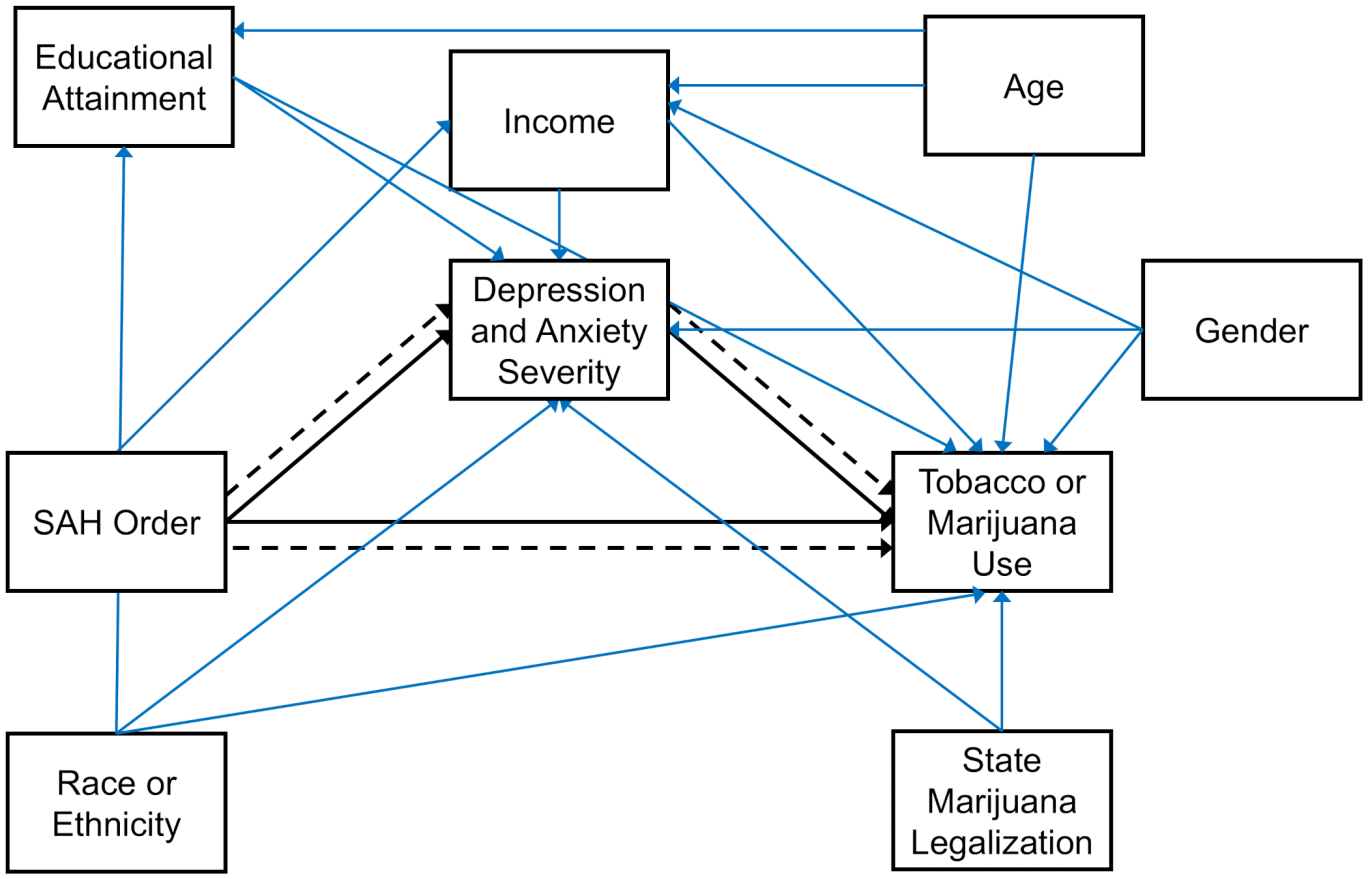
Directed Acyclic Graph (DAG) showing the proposed model. Mediation pathways are shown in black; all others are blue. Parallel hashed and solid lines indicate decomposed (between-person and within-person) effects. Not all arrows are shown to simplify the graph.

We examined how SAH orders influenced depressive and anxious symptom severity, and how symptom severity, in turn, influenced cigarette and marijuana use using two separate models: one model with cigarette use as the outcome, and the second with marijuana use as the outcome. Thus, we estimated the direct and indirect (through depression/anxiety) effects of SAH orders on cigarette and marijuana use. The relative timing of exposure, mediator, and outcome can influence the magnitude of the mediated effect [37–40]; we included a one-wave (approximately two-week) lagged indicator of SAH orders in prediction of PHQ-4 scores and cigarette and marijuana use. All analyses controlled for sex, age, race and ethnicity, educational attainment, and state marijuana legalization. Confounders were identified using a directed acyclic graph (DAG; Fig 1). No adjustments were made for multiple comparisons in this study [41,42]. Complete-case analysis was used for all analyses.

We estimated the effect of depression/anxiety on the odds of cigarette or marijuana use in two separate, simultaneously estimated survey-weighted Generalized Estimating Equations (GEE) with a logistic link function and Huber-White robust standard errors as described in previous literature [43]. We specified two regressions in which: 1) moderate/severe PHQ-4 scores were predicted by SAH orders, and 2) the outcome (cigarette or marijuana use) was predicted by SAH orders and PHQ-4 scores. We specified a first-order autoregressive working correlation matrix because: 1) the equal temporal spacing of the UAS data (biweekly), and 2) measurements taken closer in time are more likely to be related than measurements further apart.

**Fig 2:**
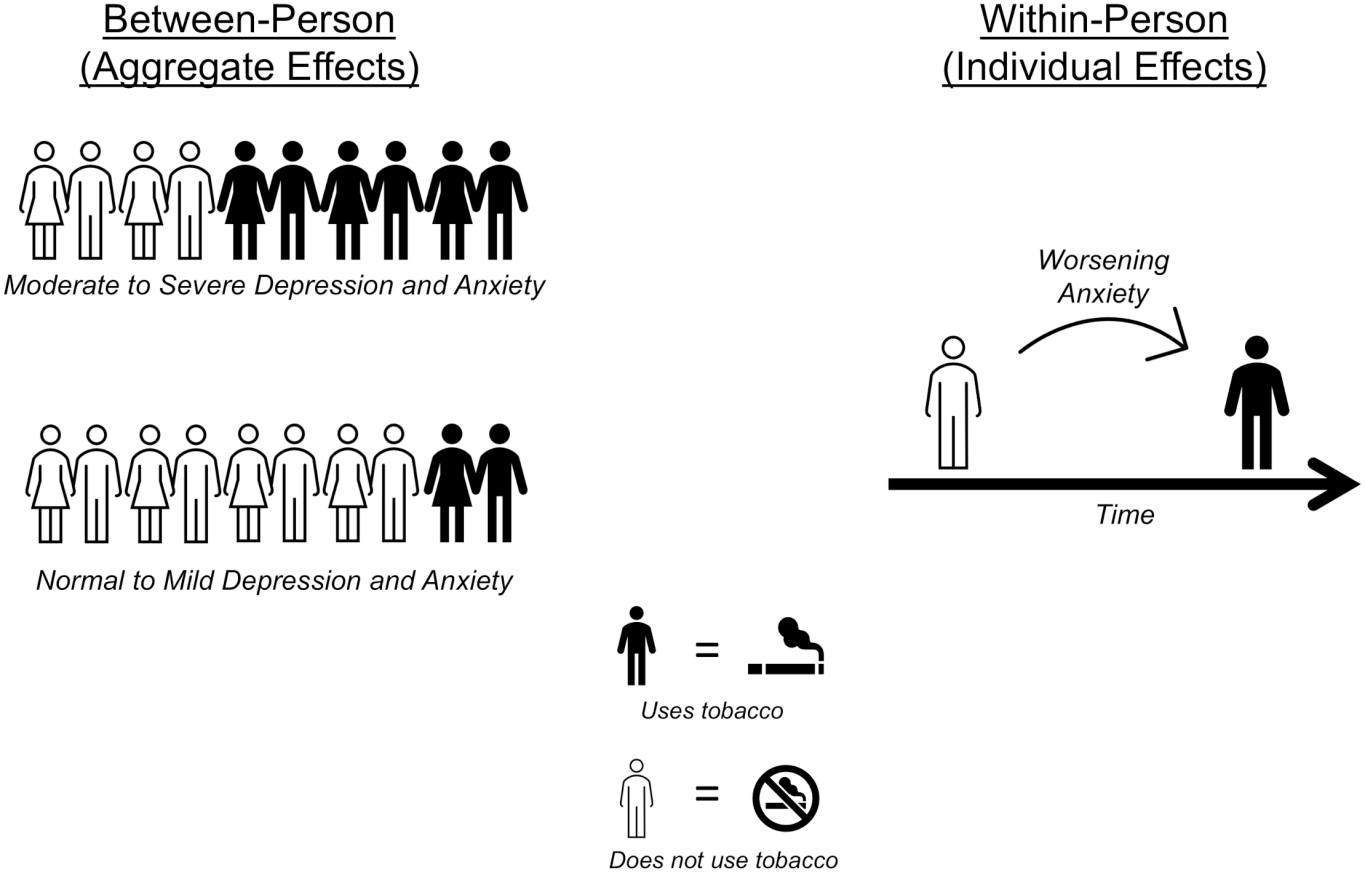
Example of the difference between-person (aggregate-level) and within-person (individual-level) effects.

There are two sources of variation within longitudinal data: between-person (aggregate level effects) and within-person (individual level effects; Fig 2). Failure to separate these two sources could result in invalid conclusions because individual and aggregated effects may suggest distinct relations with estimated outcomes (i.e., the ecological fallacy) [44]. Decomposition of between-person and within-person effects was achieved using a centering within clusters approach and including both person-means and person-mean-centered observations for categorical variables [45–47]. The inclusion of person-mean-centered observations allowed for the estimation of how changes in symptom severity influence substance use outcomes, controlling for an individual’s baseline symptom severity relative to other participants.

A mediated effect is often estimated by fitting two different models to the data in a sequential manner, assuming that the paths of the indirect effect are uncorrelated. However, these paths in nested data are correlated, requiring simultaneous estimation [43]. While the individual paths might not be significant, the indirect path itself might be significant due to non-zero correlation between the component paths. We used a Monte Carlo resampling procedure to account for this correlation [43,48] and a two-sided p-value less than 0.05 to indicate the statistical significance. All analyses were conducted in SAS (version 9.4) and R (version 4.3.1).

### Ethics statement

This research involved a secondary data analysis from the Understanding America Study. Institutional Review Board approval was not sought. The Understanding America Study underwent Institutional Review Board review at the University of Southern California.

## Results

### Descriptive statistics

Across all seven waves of the UAS survey, the prevalence of cigarette smoking was higher among those not under a SAH order (16.1% compared to 13.4%; Table 1). In contrast, the prevalence of marijuana use was slightly higher among those under a SAH order (12.6%) compared to those not (11.7%). Further, the mean PHQ-4 score across all persons was significantly higher among those under a SAH order (2.27) compared to those not (1.83).

**Table 1:** Weighted sociodemographic characteristics of the sample by exposure status: Understanding America Study (UAS) Waves 1-7.

Variable	Overall %	Under SAH %	Not Under SAH %
Under SAH	50.8	100.0	0.0
Not Under SAH	49.2	0.0	100.0
*Race*			
White, Non-Hispanic	77.1	77.0	77.1
Black, Non-Hispanic	12.6	11.1	14.1
Asian	5.4	6.7	4.2
Other*	4.9	5.2	4.6
*Ethnicity*			
Not Hispanic or Latino	83.6	81.5	85.7
Hispanic or Latino	16.4	18.5	14.3
*Gender*			
Male	48.5	49.0	47.9
Female	51.5	51.0	52.1
*Educational Attainment*			
Less than High School	8.5	8.2	8.8
High School or Equivalent	29.5	28.3	30.8
Some College	27.7	27.9	27.5
College Graduate	34.3	35.6	32.9
Cigarette Use	14.9	13.4	16.1
No Cigarette use	85.1	86.6	83.9
Marijuana Use	12.2	12.6	11.7
No Marijuana Use	87.8	87.4	88.3
Any Marijuana Legalization	70.0	76.3	63.5
No Marijuana Legalization	30.0	23.8	36.5
	Mean (SD)	Mean (SD)	Mean (SD)
Age	48.47 (.08)	48.53 (.11)	48.42 (.11)
PHQ-4 Score	2.05 (.01)	2.27 (.02)	1.83 (.02)

**Fig 3:**
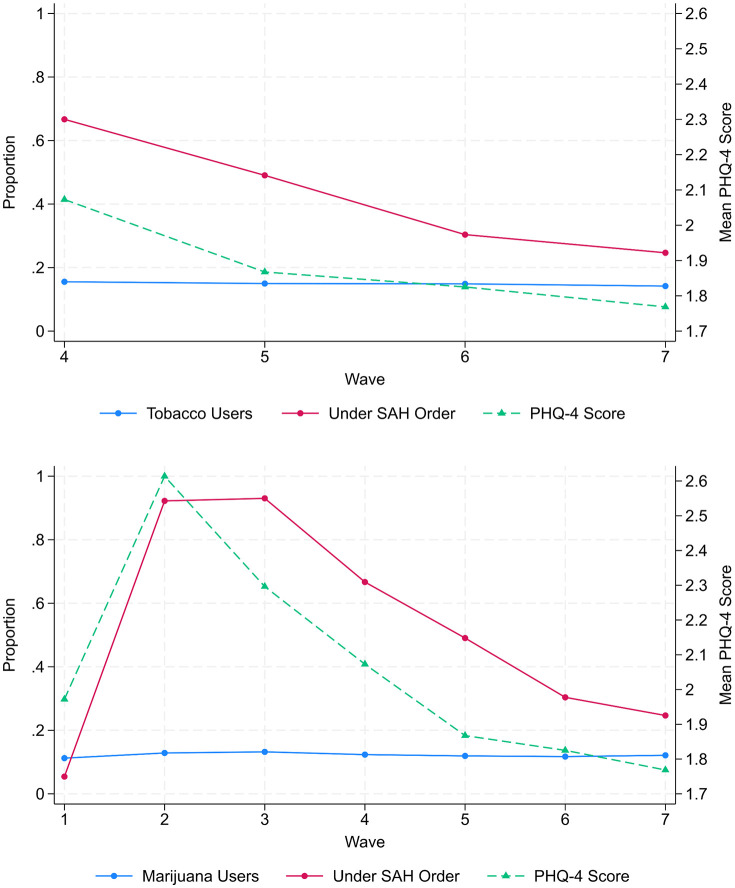
Survey weighted proportions of cigarette users (Top), marijuana users (Bottom), and persons under a Stay-At-Home order presented with survey-weighted mean PHQ-4 score by UAS wave.

During the first wave (March 10 – March 31, 2020), only 5.4% of the sample was under a SAH order. This peaked during wave three (April 15 – May 12, 2020), where 93.0% of the sample was under a SAH order (Fig 3). The prevalence of cigarette smoking consistently declined across waves four (April 29 – May 26, 2020) through seven (June 10 – July 9, 2020), from 15.5% at wave four to 14.2% at wave 7. The prevalence of marijuana use was 11.2% during the first wave, peaked at 13.2% during wave three, and declined to 12.1% at wave seven. The mean PHQ-4 score increased sharply from 1.97 during wave one to 2.61 at wave two and declined each following wave with a mean score of 1.77 at the seventh wave. Across all waves, those under a SAH order had higher mean PHQ-4 scores compared to those not (Table 2).

**Table 2:** Weighted characteristics of the sample by wave and exposure status: Understanding America Study (UAS) Waves 1- 7.

	Under SAH Order			Not Under SAH Order		
Survey Wave*	Mean PHQ-4 Score	Proportion Cigarette Use** (%)	Proportion Cannabis Use (%)	Mean PHQ-4 Score	Proportion Cigarette Use** (%)	Proportion Cannabis Use (%)
Wave 1	2.90	—	14.1	1.92	—	11.1
Wave 2	2.63	—	12.6	2.42	—	16.4
Wave 3	2.32	—	13.2	1.93	—	14.0
Wave 4	2.12	14.5	12.2	1.98	17.6	12.2
Wave 5	1.98	13.7	11.8	1.76	16.2	12.0
Wave 6	1.99	12.1	11.8	1.75	16.1	11.7
Wave 7	2.10	11.0	13.7	1.66	15.2	11.6

*Survey wave indicates the prespecified dates when surveys were sent to participants. Dates of each survey wave are as follows: Wave 1 (March 10–31, 2020); Wave 2 (April 1–14, 2020); Wave 3 (April 15 – May 12, 2020); Wave 4 (April 29 – May 26, 2020); Wave 5 (May 13 – June 9, 2020); Wave 6 (May 27 – June 23, 2020); Wave 7 (June 10 – July 9, 2020).

**Cigarette use was not assessed during the UAS survey until wave 4.

### Effect of COVID-19 SAH orders on PHQ-4 scores (waves 1–7)

Across the first seven waves of the UAS COVID-19 tracking survey, age, higher educational attainment, and Black race were associated with lower odds of moderate-to-severe PHQ-4 scores (Table 3). There was no evidence of differences in odds of PHQ-4 severity among other racial groups, compared to white respondents. Female respondents had 1.66 (95% CI: 1.38, 1.98) times the odds of elevated depressive or anxious symptoms, relative to males. Compared to persons not under a SAH order, those under a SAH order had higher odds of moderate-to-severe depression and anxiety (OR = 2.18; 95% CI: 1.27, 3.73). However, the relaxation of a SAH order within a state did not influence an individual’s mental health symptoms.

**Table 3:** Odds of moderate/severe PHQ-4 scores across the first seven waves of the UAS COVID-19 tracking survey (marijuana model).

	OR	95% CI	p-value
**Between Person**			
Under SAH Order	2.18	(1.27, 3.73)	.0046
Age	0.98	(0.97, 0.98)	<.0001
*Gender*			
Male	REF	—	—
Female	1.66	(1.38, 1.98)	<.0001
*Race*			
White, non-Hispanic	REF	—	—
Black, non-Hispanic	0.65	(0.47, 0.88)	.0059
Asian	0.92	(0.63, 1.33)	.6489
Other	1.03	(0.71, 1.49)	.8940
*Ethnicity*			
Not Hispanic or Latino	REF	—	—
Hispanic or Latino	0.81	(0.61, 1.07)	.1415
*Educational Attainment*			
Less than High School	REF	—	—
High School or GED	0.66	(0.49, 0.89)	.0069
Some College	0.64	(0.48, 0.85)	.0024
College Graduate	0.56	(0.42, 0.76)	.0001
**Within Person**			
Under SAH Order	0.96	(0.87, 1.05)	.3690

### Effect of COVID-19 SAH orders on PHQ-4 scores (waves 4–7)

As cigarette use was only included in UAS waves 4–7, the model with cigarette use as the outcome only included data from these waves. Increases in age, higher educational attainment, female gender, and Black race were associated with lower odds of moderate-to-severe PHQ-4 scores (Table 4). As seen in the model with all seven waves, for between-person effects, those under a SAH order had higher odds of moderate or severe mental health symptoms (OR = 2.21; 95% CI: 1.16, 4.21) compared to those not under a SAH order. There was no evidence that the relaxation of SAH orders influenced depression/anxiety.

**Table 4:** Odds of moderate/severe PHQ-4 scores across waves four through seven of the UAS COVID-19 tracking survey (cigarette model).

	OR	95% CI	p-value
**Between Person**			
Under SAH Order	2.21	(1.16, 4.21)	.0163
Age	0.98	(0.97, 0.98)	<.0001
*Gender*			
Male	REF	—	—
Female	1.73	(1.41, 2.12)	<.0001
*Race*			
White, non-Hispanic	REF	—	—
Black, non-Hispanic	0.68	(0.48, 0.96)	.0288
Asian	0.95	(0.63, 1.45)	.8274
Other	0.99	(0.65, 1.51)	.9664
*Ethnicity*			
Not Hispanic or Latino	REF	—	—
Hispanic or Latino	0.86	(0.63, 1.16)	0.3232
*Educational Attainment*			
Less than High School	REF	—	—
High School or GED	0.69	(0.49, 0.98)	.0358
Some College	0.63	(0.45, 0.88)	.0066
College Graduate	0.59	(0.42, 0.84)	.0030
**Within Person**			
Under SAH Order	1.13	(0.97, 1.32)	.1202

### Effects of COVID-19 SAH orders and PHQ-4 scores on marijuana use

There was no evidence of a between-person effect of SAH orders on current marijuana use, comparing those under a SAH order to those not and controlling for covariates and PHQ-4 scores (Table 5). There was no evidence of an association between the relaxation of SAH orders and an individual’s current marijuana use. The odds of current marijuana use for persons with depressive/anxious symptoms were 0.37 (95% CI: 0.17, 0.84) times that of persons with normal-to-mild symptoms. Within a person, the odds of persons with depressive/anxious symptoms using marijuana were 0.22 (95% CI: 0.12, 0.40) times that of persons with milder depressive/anxious symptoms. Higher age, female gender, higher educational attainment, and Asian race were associated with lower odds of marijuana use. Living in a state with any form of legalized marijuana use was associated with increased odds of marijuana use (OR = 1.37; 95% CI: 1.11, 1.68).

**Table 5:** Odds of current marijuana use across the first seven waves of the UAS COVID-19 tracking survey.

	OR	95% CI	p-value
***Direct Effects***			
**Between Person**			
Under SAH Order	2.04	(1.08, 3.86)	.0281
Moderate/Severe PHQ-4	0.37	(0.17, 0.84)	.0167
Age	0.97	(0.97, 0.98)	<.0001
*Gender*			
Male	REF	—	—
Female	0.74	(0.62, 0.90)	.0021
*Race*			
White, non-Hispanic	REF	—	—
Black, non-Hispanic	1.27	(0.96, 1.69)	.0946
Asian	0.42	(0.25, 0.73)	.0018
Other	1.44	(1.01, 2.05)	.0458
*Ethnicity*			
Not Hispanic or Latino	REF	—	—
Hispanic or Latino	0.76	(0.57, 1.01)	.0603
*Educational Attainment*			
Less than High School	REF	—	—
High School or GED	0.68	(0.49, 0.95)	.0255
Some College	0.69	(0.50, 0.96)	.0258
College Graduate	0.46	(0.33, 0.65)	<.0001
No Marijuana Legalization	REF	—	—
Marijuana Legalization	1.37	(1.11, 1.68)	.0027
**Within Person**			
Under SAH Order	0.96	(0.87, 1.06)	.3963
Moderate/Severe PHQ-4	0.22	(0.12, 0.40)	<.0001
	$e^{\hat{ab}}$	95% CI	p-value
***Indirect Effects***			
Between Person SAH Order; Between Person PHQ-4 scores	0.46	(0.35, 0.60)	<.01*
Between Person SAH Order; Within Person PHQ-4 scores	0.31	(0.21, 0.44)	<.01*
Within Person SAH Order; Between Person PHQ-4 scores	1.04	(0.97, 1.12)	>.05*
Within Person SAH Order; Within Person PHQ-4 scores	1.07	(0.96, 1.19)	>.05*

*Exact p-values for indirect effects were not calculated. Confidence intervals generated at α = .05 and α = .01 were used to determine significance.

### Mediated effects of COVID-19 SAH orders on marijuana use through PHQ-4 scores

In the first path of the mediated effect, there was a significant between-person effect of SAH orders on PHQ-4 scores (OR = 2.18, 95% CI: 1.27, 3.73); relaxation of SAH orders on PHQ-4 scores was not significant. In the second path of the mediated effect, there were between- and within-person effects of PHQ-4 scores on marijuana use. In terms of between-person effects, the odds of a person using marijuana were 0.37 (95% CI: 0.17, 0.84) times that of a person not experiencing moderate-to-severe anxiety or depression. In terms of within-person effects, the odds of using marijuana for a person who was anxious or depressed were 0.22 (95% CI: 0.12, 0.40) times that of a person who was not experiencing moderate-to-severe depression or anxiety. Taken together, there was a significant indirect effect of between-person SAH on marijuana use through between-person PHQ-4 scores ($e^{\hat{ab}}$ = 0.46, 95% CI: 0.35, 0.60). There was also a significant indirect effect of between-person SAH on marijuana use through within-person PHQ-4 scores ($e^{\hat{ab}}$ = 0.31, 95% CI: 0.21, 0.44). SAH orders tended to increase the odds of increased depression/anxiety, but increased depression/anxiety (at both between- and within-person levels) decreased marijuana smoking.

Mediated effects suggest an inverse relationship between SAH orders and current marijuana use through PHQ-4 symptoms, where SAH orders correspond with lower odds of current marijuana use. While the mediated effects consisting of the between-person effects of SAH orders on marijuana use were significant, there was no evidence of a mediated effect comprised by the within-person changes in SAH order status.

### Effects of COVID-19 SAH orders and PHQ-4 scores on cigarette use

There was no evidence of between-person differences in SAH order exposure influencing cigarette use, after controlling for covariates and PHQ-4 severity (Table 6). The relaxation of SAH orders was not associated with current cigarette use. Compared to those with normal-to-mild PHQ-4 scores, those with elevated symptoms had lower odds of cigarette smoking (OR = 0.29; 95% CI: 0.13, 0.65). Within a person, shifting from normal to moderate-severe PHQ-4 scores was associated with 0.26 (95% CI: 0.15, 0.47) times the odds of cigarette smoking. Increased age, higher educational attainment, and Asian race were inversely associated with cigarette use (Table 6). There was no evidence that marijuana legalization was associated with current cigarette use.

**Table 6:** Odds of current cigarette use during the first seven waves of the UAS COVID-19 tracking survey.

	OR	95% CI	p-value
***Direct Effects***			
**Between Person**			
Under SAH Order	0.83	(0.43, 1.60)	.5687
Moderate/Severe PHQ-4	0.29	(0.13, 0.65)	.0026
Age	0.99	(0.98. 0.99)	.0001
*Gender*			
Male	REF	—	—
Female	0.92	(0.77, 1.11)	.3879
*Race*			
White, non-Hispanic	REF	—	—
Black, non-Hispanic	1.08	(0.81, 1.44)	.5802
Asian	0.53	(0.31, 0.90)	.0183
Other	0.73	(0.48, 1.11)	.1376
*Ethnicity*			
Not Hispanic or Latino	REF	—	—
Hispanic or Latino	.63	(.46,.86)	.0043
*Educational Attainment*			
Less than High School	REF	—	—
High School or GED	0.41	(0.30, 0.56)	<.0001
Some College	0.29	(0.21, 0.39)	<.0001
College Graduate	0.10	(0.07, 0.13)	<.0001
No Marijuana Legalization	REF	—	—
Marijuana Legalization	1.16	(0.95, 1.41)	.1547
**Within Person**			
Under SAH Order	0.93	(0.83, 1.06)	.2780
Moderate/Severe PHQ-4	0.26	(0.15, 0.47)	<.0001
	$e^{\hat{ab}}$	95% CI	p-value
***Indirect Effects***			
Between Person SAH Order; Between Person PHQ-4 scores	0.37	(0.24, 0.55)	<.01*
Between Person SAH Order; Within Person PHQ-4 scores	0.35	(0.22, 0.53)	<.01*
Within Person SAH Order; Between Person PHQ-4 scores	0.86	(0.75, 0.97)	<.05*
Within Person SAH Order; Within Person PHQ-4 scores	0.85	(0.74, 0.97)	<.05*

*Exact p-values for indirect effects were not calculated. Confidence intervals generated at α = .05 and α = .01 were used to determine significance.

### Mediated effects of COVID-19 SAH orders on cigarette use through PHQ-4 scores

In the first path of the mediated effect, there was a significant between-person effect of SAH orders on PHQ-4 scores (OR = 2.21; 95% CI: 1.16, 4.21). Relaxation of SAH orders was not associated with depressive or anxious symptoms. In the second path of the mediated effect, there were both between- and within-person effects of PHQ-4 scores. Compared to those with mild-to-normal symptoms, those with moderate-to-severe symptoms had 0.29 (95% CI: 0.13, 0.65) times the odds of cigarette use. Within a person, the odds of current cigarette use for someone with moderate-to-severe depression or anxiety was 0.27 (95% CI: 0.15, 0.47) times that of the same person with normal-to-mild symptoms. These findings indicate a significant indirect effect of between-person SAH orders on cigarette smoking through between-person changes in PHQ-4 scores ($e^{\hat{ab}}\;$= 0.37; 95% CI: 0.24, 0.55). There was also a significant indirect effect comprised of between-person SAH order on cigarette use and within-person changes in PHQ-4 scores ($e^{\hat{ab}}$ = 0.35; 95% CI: 0.22, 0.53).

Tests of mediated effects suggest an inverse relationship between SAH orders and cigarette smoking through depressive or anxious symptoms. Mediated effects suggest that SAH orders correspond with lower odds of current cigarette use. Though both mediated effects comprised by state-level changes in SAH orders were significant, caution should be taken when interpreting these, considering one of the component paths was not statistically significant (Table 6).

## Discussion

We found evidence that the effect of COVID-19 SAH orders on cigarette and marijuana use was mediated by PHQ-4 scores. For both cigarette and marijuana use, our results suggest that exposure to a SAH order (between-person) resulted in increased odds of moderate-to-severe depression or anxiety symptoms and that experiencing moderate or severe mental health symptoms (between- and within-persons) significantly lowered the odds of using either cigarette or marijuana use.

Longitudinal studies of marijuana use suggest no appreciable change during the pandemic [29,49,50]. Changes in tobacco use during the pandemic have been mixed, with some studies suggesting increasing smoking, and others suggesting either a decline or no change [25,51–57]. Our results indicate that across the first seven waves, there was no substantive change in the point-prevalence of marijuana use (Fig 3). There was also no noticeable change in the point-prevalence of cigarette use across waves four through seven. Prior evidence notes that persons with moderate-to-severe depression or anxiety exhibited greater tobacco or marijuana use during the pandemic, which does not align with our findings [58–62]. However, our results are consistent with literature suggesting that COVID-19 SAH orders were associated with worsening mental health symptoms [27,51,63,64].

Several factors may explain the discrepancy between our findings and prior literature. First, many prior studies relied on cross-sectional studies, which significantly limit inference on the temporal ordering of mental health and substance use in response to the pandemic. Our longitudinal framework, including the implementation of temporally lagged predictors, may better capture dynamic responses to SAH orders. Second, the divergence in findings could reflect differences in sample characteristics. Some of the prior research that highlighted increased marijuana use during the pandemic focused on specific populations, such as gender and sexual minorities [65], youth and young adults [66], or a substance use history. Third, our measurement of marijuana or cigarette use reflected the past week of use, which could have been more sensitive to short-term behavior or mental health changes. Finally, mixed findings of the impact of COVID-19 on tobacco and marijuana in prior research could be attributable to the heterogeneous policy environment created by the pandemic. as there was notable geographic variation in policy implementation and efficacy [67–69].

During the pandemic, economic concerns were one of the strongest predictors of poor mental health [70]. Prior economic literature on recessions suggests that worsened economic conditions contribute to increased propensity to use substances, including tobacco and marijuana [71–73]. Conversely, the framework of an “inhibition effect,” which comes from the economics literature, posits that persons living in adverse economic conditions become more risk-averse and the macroecological conditions “inhibit” aberrant or deviant behaviors [74]. The finding that recessions have been associated with increased healthy behaviors and lower mortality supports this “inhibition effect” [22–24,75].

Extending this framework to the COVID-19 pandemic, wherein SAH orders act as an ecological stressor akin to concerns from a contracting economy, implies that persons with heightened depression or anxiety increase their self-regulatory behavior and reduce substance use. “Inhibition effects” in responses to non-economic ecological exposures are not unusual. During the H1N1 pandemic, persons more aware of the risks of disease were more willing to engage in protective health-related behaviors [76]. A longitudinal study of tobacco users concluded that those who quit and remained tobacco-free had an elevated perceived-risk of health complications from COVID-19 due to smoking [77]. It is possible that either economic concerns or health concerns would be motivating persons to quit using substances. We did not directly assess these hypotheses.

It is plausible that the consequences of pandemic-related job loss, such as changes in income or disruptions in health care access, may have acted as a confounder in the association between SAH orders, mental health, and substance use. The COVID-19 pandemic was associated with both job loss and increased psychological distress [78,79]. Research before the COVID-19 pandemic suggests that involuntary job loss, such as that experienced due to the pandemic, may slightly increase the probability of smoking [80,81]. Simultaneously, job loss can lead to the loss of employer-sponsored health insurance, which could have limited access to mental health services. Despite public assistance programs, many Americans lost health insurance access due to job loss during COVID-19 [82]. However, government stimulus checks and benefits provided during the pandemic, such as expansions in unemployment benefits, may have mitigated the impact on both economic concerns and psychological distress that have been observed in economic contractions before COVID-19 [83,84]. Income and employment information were included in the “My Household” section of the UAS survey and were only required to be updated quarterly. Due to potential temporal misclassification of when changes occurred relative to assessments of variables of interest, this was not assessed as a potential mediating mechanism.

A separate hypothesis that may partially explain our findings is that social isolation, as a result of SAH orders, reduced tobacco and marijuana use. Persons consuming tobacco or marijuana only in social contexts may have had reduced opportunities to use. There is evidence of reduced cigarette smoking among persons who identify as “social smokers” [85]. This phenomenon may extend to marijuana use.

These findings have important implications for public health and policy responses in future pandemics or population-level events such as economic recessions or natural disasters. Policies such as SAH orders, which were implemented to reduce the spread of a novel disease, may have unintended psychological consequences. Interestingly, our findings suggest that heightened psychological distress may reduce, rather than increase, substance use. However, we did not assess other potential psychological sequelae of increased psychological distress, such as increased suicidal ideation. Increasing access to mental health services, particularly in rural areas, may be an effective measure to support population well-being in the event of future public health emergencies.

### Strengths and limitations

The strengths of the study include the use of a longitudinal nationally representative dataset with consistent survey methodology during the COVID-19 pandemic. Using a longitudinal dataset, we were able to assess a mediation model for the effect of COVID-19 SAH orders on current cigarette and marijuana use. Further, we used a clinically validated scale of depressive and anxious symptoms.

Studies that assess the effect of an ecological exposure are often viewed with concern due to the potential for misinterpreting population-level effects as individual-level effects (the ecological fallacy). To mitigate this, we decomposed all variables in our model into mutually independent between-person and within-person effects. Despite this, using a GEE model to estimate these effects requires noting that all reported effects are population-averaged effects.

One limitation with our study design is that SAH orders were measured at the state level, and our analysis assumes that individuals experienced SAH orders homogenously within a state. It is possible that individuals living in rural areas, compared to urban areas, may have experienced SAH orders differently due to population density or variations in policy enforcement. Due to limitations in the UAS data, rural-urban status could not be included in the analyses. A related limitation is that we could not assess compliance with SAH policies. It is possible that adherence to COVID-19 policies, rather than the presence of the policies themselves, could explain some of our findings. The phenomenon of “COVID fatigue,” where compliance with policy restrictions on individual behaviors decreased over time, could have confounded our results [86]; however, we restricted our analysis to surveys completed prior to July 9, 2020, which may mitigate the influence of “COVID fatigue”.

A key limitation is that we use self-reported data rather than objective or biochemical verification of substance use status. It is possible that our results are based on an underreporting of substance use, but there is no reason to hypothesize that the validity of self-reported measures changed dramatically during the COVID-19 pandemic.

Further, we were restricted to defining temporal relationships in our mediation model by the two-week gaps between waves. Our model assumes a two-week lagged effect for SAH orders on mental health symptoms and cigarette or marijuana use. These may not be representative of the true temporal mechanism. Due to limitations in our model, we dichotomized PHQ-4 scores. Unfortunately, this resulted in the loss of granular information from the original PHQ-4 scale. Hierarchical mediation models using generalized structural equation modeling remain an active area of current research; the use of such a model may address limitations in our analytic approach.

Future prospective-cohort studies could build on our findings through the use of data sources with greater temporal and geographic granularity to capture individual-level variations in policy exposure and compliance. Additionally, ecological momentary assessment (EMA) could track changes in mood and behavior in real-time. Further, the implementation of biochemical verification of tobacco or marijuana use, such as saliva cotinine or urine cannabinoid screening, could improve the validity of self-reported measures.

### Conclusions

Overall, we found evidence that SAH orders had a detrimental effect on depression and anxiety, with SAH orders being associated with increased symptom severity. Further, we found that worsened between- and within-person depressive and anxious symptoms were strongly predictive of lower cigarette and marijuana use. Taken together, we found evidence that moderate-to-severe depressive and anxious symptoms mediated the relationship between SAH orders and both cigarette and marijuana use. In other words, the mitigative effect of SAH orders on cigarette and marijuana use can be explained by the influence of SAH orders on depression and anxiety. Our findings of elevated PHQ-4 scores being predictive of lower odds of tobacco and marijuana use were inconsistent with expectations using the self-medication hypothesis as a guiding framework. Future studies may assess whether our observed phenomenon is unique to the post-COVID period or is representative of a larger mechanism in response to population-level stressors. We contribute to the growing literature on the mechanisms by which ambient ecological stressors impact mental health and substance use.
